# Aryl Hydrocarbon Receptor Repressor and TiPARP (ARTD14) Use Similar, but also Distinct Mechanisms to Repress Aryl Hydrocarbon Receptor Signaling

**DOI:** 10.3390/ijms15057939

**Published:** 2014-05-06

**Authors:** Laura MacPherson, Shaimaa Ahmed, Laura Tamblyn, Jean Krutmann, Irmgard Förster, Heike Weighardt, Jason Matthews

**Affiliations:** 1 Department of Pharmacology and Toxicology, University of Toronto, Toronto, ON M5S 1A8, Canada; E-Mails: l.macpherson@mail.utoronto.ca (L.M.); shaimaa.ahmed@utoronto.ca (S.A.); laura.tamblyn@utoronto.ca (L.T.); 2 IUF-Leibniz Research Institute for Environmental Medicine gGmbH, Auf’m Hennekamp 50, 40225 Düsseldorf, Germany; E-Mail: krutmann@uni-duesseldorf.de; 3 Immunology and Environment, Life and Medical Sciences (LIMES) Institute, University of Bonn, Carl-Troll-Straβe 31, 53115 Bonn, Germany; E-Mails: irmgard.foerster@uni-bonn.de (I.F.); hwei@uni-bonn.de (H.W.)

**Keywords:** aryl hydrocarbon receptor, AHR (Aryl hydrocarbon receptor) repressor, TCDD (2,3,7,8-tetrachlorodibenzo-*p*-dioxin)-inducible-poly (ADP-ribose) polymerase, ADP-ribosyltransferase diptheria-like toxin 14, 2,3,7,8-tetrachlorodibenzo-*p*-dioxin, transactivation

## Abstract

The aryl hydrocarbon receptor (AHR) regulates the toxic effects of 2,3,7,8-tetrachlorodibenzo-*p*-dioxin (TCDD). The AHR repressor (AHRR) is an AHR target gene and functions as a ligand-induced repressor of AHR; however, its mechanism of inhibition is controversial. Recently, we reported that TCDD-inducible poly (ADP-ribose) polymerase (TiPARP; ARTD14) also acts as a repressor of AHR, representing a new player in the mechanism of AHR action. Here we compared the ability of AHRR- and TiPARP-mediated inhibition of AHR activity. TCDD increased AHRR mRNA levels and recruitment of AHRR to *cytochrome P450 1A1* (*CYP1A1*) in MCF7 cells. Knockdown of TiPARP, but not AHRR, increased TCDD-induced CYP1A1 mRNA and AHR protein levels. Similarly, immortalized *TiPARP*^−/−^ mouse embryonic fibroblasts (MEFs) and *AHRR*^−/−^ MEFs exhibited enhanced AHR transactivation. However, unlike *TiPARP*^−/−^ MEFs, *AHRR*^−/−^ MEFs did not exhibit increased AHR protein levels. Overexpression of TiPARP in *AHRR*^−/−^ MEFs or AHRRΔ8, the active isoform of AHRR, in *TiPARP*^−/−^ MEFs reduced TCDD-induced CYP1A1 mRNA levels, suggesting that they independently repress AHR. GFP-AHRRΔ8 and GFP-TiPARP expressed as small diffuse nuclear foci in MCF7 and HuH7 cells. GFP-AHRRΔ8_Δ1-49, which lacks its putative nuclear localization signal, localized to both the nucleus and the cytoplasm, while the GFP-AHRRΔ8_Δ1-100 mutant localized predominantly in large cytoplasmic foci. Neither GFP-AHRRΔ8_Δ1-49 nor GFP-AHRRΔ8_Δ1-100 repressed AHR. Taken together, AHRR and TiPARP repress AHR transactivation by similar, but also different mechanisms.

## 1. Introduction

Aryl hydrocarbon (AHR) is a ligand-activated transcription factor and a member of the basic-helix-loop-helix Per (Period)-ARNT (aryl hydrocarbon receptor nuclear translocator)-SIM (single-minded) (bHLH/PAS) protein superfamily. AHR mediates the toxic effects of the environmental contaminant, 2,3,7,8-tetrachlorodibenzo-*p*-dioxin (TCDD), but it is also activated by numerous endogenous, natural and dietary compounds [1]. Despite extensive research, the biological role of AHR remains unclear. However, there is significant evidence to suggest that AHR plays important roles in growth, differentiation, reproduction, immunity, and the vasculature [2]. Once activated by the ligand, AHR translocates from the cytoplasm into the nucleus where it associates with its obligatory heterodimerization partner, ARNT. The activated AHR/ARNT complex binds to AHR response elements (AHREs) located within the regulatory regions of AHR target genes such as *cytochrome P450 1A1* (*CYP1A1*), *CYP1B1*, *AHR repressor (AHRR) and TCDD-inducible poly-ADP-ribose polymerase* [*TiPARP*]) also known as *ADP-ribosyltranferase diphtheria-like toxin 14 (ARTD14)* [3–5]. After binding to DNA, the AHR recruits a variety of different coregulatory proteins resulting in changes in target gene expression [6].

The mechanism by which AHR regulates its target genes is for the most part well understood; however, our understanding of how a transcriptionally activated AHR is regulated or inhibited is incomplete. Some of the proposed mechanisms include: (1) increased metabolism of the activating ligand through induction of drug metabolizing enzymes; (2) ligand-induced proteolytic degradation of AHR via the ubiquitin/proteasome pathway; and (3) negative auto-regulatory feedback control via the induction of AHR-dependent repressors, such as AHRR [3,7,8]. The AHRR, which is also a member of the bHLH protein family, is structurally similar to AHR but lacks a ligand-binding region (PAS B domain) and an activation domain [4]. Two AHRR splicing variants have been described in immortalized human cell lines, full length AHRR_719_ (amino acids 1-719; NM_020731) and AHRRΔ8 (amino acids 1-701; NM_001242412) with lacks exon 8 [9]. AHRRΔ8 is the active and the predominant isoform in human cell lines [9]. Much of the current understanding of AHRR-mediated repression of AHR is based on *in vitro* transient transfection and overexpression experiments [4,9]. Under these conditions, AHRR strongly inhibits AHR transactivation, but its mechanism of inhibition remains unclear. It was first proposed that AHRR repressed AHR through competition between AHR/ARNT and AHRR/ARNT complexes for binding to AHREs [4]. However, overexpression of ARNT fails to rescue AHRR-dependent repression of AHR and mutation of the DNA binding domain does not affect the ability of AHRR to repress AHR [9,10]. Moreover, AHRR expression levels do not correlate with CYP1 activity in human skin fibroblasts nor do they correlate with CYP1A1 responsiveness in mice exposed to the AHR agonist, benzo[*a*]pyrene (B[*a*]P) [11,12]. Treatment of *AHRR*^−/−^ mice with the AHR agonist, 3-methylcholanthrene, results in tissue-specific rather than whole body increases in CYP1A1 mRNA levels [13]. Collectively, these data suggest that the current model of AHRR-mediated repression is not fully understood and that alternative mechanisms to inhibit AHR exist.

ADP-ribosyltransferases (ARTs), formerly known as poly(ADP-ribose) polymerases [PARPs], catalyze the transfer a single or multiple ADP-ribose units from nicotinamide adenine dinucleotide (NAD^+^) onto themselves or other protein substrates [14]. We recently reported that TiPARP (also known as ARTD14) is a mono-ADP-ribosyltransferase and a ligand-induced negative regulator of AHR transactivation that may play a role in regulating AHR protein levels [15,16]. Our findings revealed that similar to AHRR, TiPARP is also part of an auto-regulatory negative feedback loop regulating AHR activity. However, it remains to be determined whether AHRR and TiPARP work together, independently or in opposition to repress AHR activity.

In the present study, we compared the ability of AHRR and TiPARP to repress *in vitro* AHR activity. Our data reveal some important similarities in the actions of AHRR and TiPARP, but also demonstrate that these proteins inhibit AHR activity by distinct mechanisms.

## 2. Results and Discussion

### 2.1. Differential TCDD (2,3,7,8-Tetrachlorodibenzo-p-dioxin)-dependent Regulation of TiPARP and AHRR (Aryl Hydrocarbon Repressor) Expression

**Figure 1.**
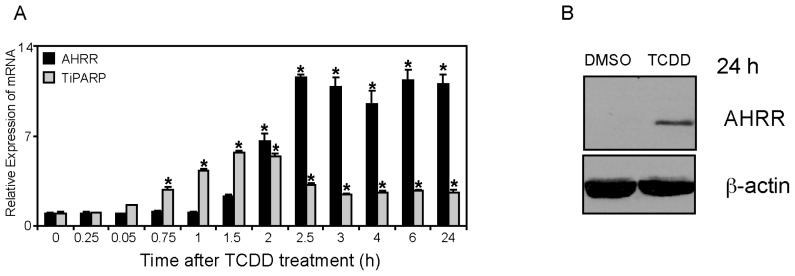
Time- and TCDD (2,3,7,8-tetrachlorodibenzo-*p*-dioxin)-dependent regulation of AHRR (Aryl hydrocarbon receptor repressor) and TiPARP mRNA levels. (**A**) MCF7 cells were treated with 10 nM TCDD at the indicated times. TiPARP and AHRR mRNA levels were determined as described in Materials and Methods. Data were presented as means ± SEM mRNA expression significantly greater (*p* < 0.05) than 0 h is denoted with an asterisk; (**B**) TCDD-induced AHRR protein expression after 24 h treatment with 10 nM TCDD. A representative Western blot from three independent experiments was provided.

AHRR and TiPARP (ARTD14) are both TCDD-responsive genes [4,16–18]; however, direct comparisons of the time-dependent regulation of these genes by TCDD has not been reported. To this end, we treated MCF7 human breast cancer cells with 10 nM TCDD for 15 min to 24 h. We chose MCF7 cells because they express functional AHR, ARNT, and AHRR and they are widely used to study AHR signaling. TCDD-dependent increases in TiPARP mRNA levels were rapid, reaching statistical significance after 45 min, peaking after 1.5 h and declining thereafter, although levels remained elevated above time zero for the remainder of the time course (Figure 1A; [15]). Significant increases in AHRR mRNA levels occurred after 2 h of TCDD treatment, reaching a maximum after 2.5 h, which was maintained throughout the time course (Figure 1A). Despite increases in AHRR mRNA levels as early as 2 h, TCDD-dependent increases in AHRR protein levels were not detectable before 24 h treatment (Figure 1B). Endogenous TiPARP protein levels were not determined due to the lack of a suitable antibody. In our hands, existing commercially available antibodies detect overexpressed TiPARP, but we have been unable to detect endogenous protein using these antibodies (data not shown). The time course data show that TiPARP mRNA levels are induced more rapidly than those of AHRR. Unlike the decline in TiPARP mRNA levels after 2 h, AHRR mRNA levels are maintained at maximum levels from 2.5 h through 24 h, suggesting distinct mechanism of regulation. Characterization of the murine AHRR 5′-upstream regulatory region revealed a tata-less promoter in which its high level of induction by AHR ligands was regulated by 3 AHREs and a GC-rich sequence [19]. The isolated human AHRR promoter region also contains multiple AHREs and is very responsive to TCDD [20]. Little is known about the AHR-dependent regulation of the TiPARP upstream regulatory region. The isolated mouse TiPARP promoter was unresponsive to TCDD, which might be due to its high constitutive expression levels [21]. These findings might also suggest a more complex mechanism of regulation of *TiPARP*. ChIP-Sequencing data, however, confirm the TCDD-dependent recruitment of AHR to the upstream regulatory regions of *AHRR* and *TiPARP* [22]. TiPARP is also induced by platelet derived growth factor, estrogen receptor alpha (ERα) and glucocorticoid receptor [23–25], suggesting that TiPARP might also influence the regulation of these receptor pathways. Similarly, AHRR has also been reported to repress hypoxia inducible factor 1α (HIF-1α) and ERα [9,26], revealing that its repressor activity and that of TiPARP extend beyond simply targeting AHR.

### 2.2. AHR (Aryl Hydrocarbon Receptor) but not ARNT (Aryl Hydrocarbon Receptor Nuclear Translocator) Overexpression Rescues AHRR-Mediated Repression of CYP1A1 Reporter Gene Activity

**Figure 2.**
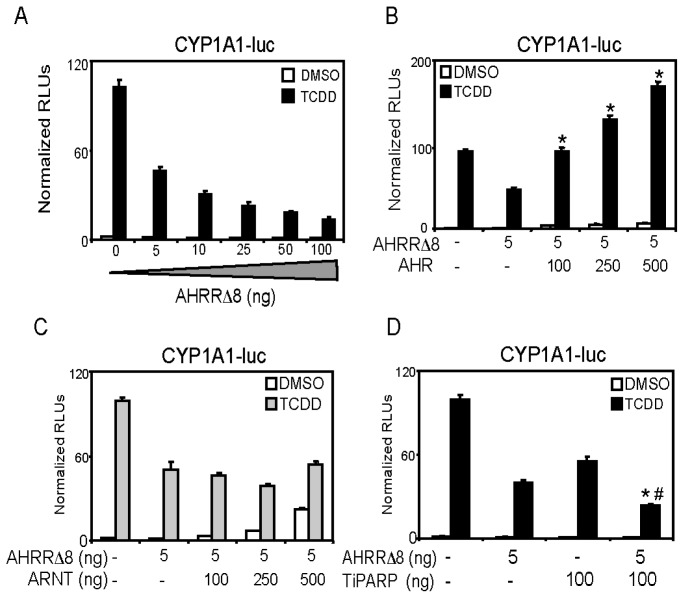
AHRRΔ8 or TiPARP repress CYP1A1-regulated reporter gene activity. HuH7 cells were transfected with pCYP1A1-luc and increasing amounts of (**A**) pcDNA-AHRRΔ8. Cells were then treated with TCDD for 24 h and reporter gene activity was determined; Overexpression of (**B**) AHR (Aryl hydrocarbon receptor); but not (**C**) ARNT (aryl hydrocarbon receptor nuclear translocator) rescued CYP1A1-luc activity; Reporter gene activity significantly different (*p* < 0.05) than cells transfected with AHRRΔ8 alone is denoted with an asterisk and (**D**) Overexpression of AHRRΔ8 and TiPARP resulted in greater repression of AHR regulated reporter gene activity than either one alone. Data are presented as means ± SEM of three independent replicates. Reporter gene activity of AHRRΔ8 + TiPARP transfected cells significantly less (*p* < 0.05) than cells transfected AHRRΔ8 or TiPARP alone was denoted with an asterisk or pound sign, respectively.

We next confirmed the ability of overexpressed AHRR to repress CYP1A1 reporter gene activity, and determined if overexpressed AHR or ARNT prevented AHRR-mediated repression of AHR. Increasing concentrations of AHRRΔ8 repressed CYP1A1 reporter gene expression (Figure 2A). AHRR_719_ failed to repress CYP1A1 reporter gene expression even when transfected with 1 μg total DNA concentration (data not shown), supporting the notion that AHRRΔ8 is the active form of AHRR in human cell lines [9]. Increasing amounts of AHR, but not ARNT prevented AHRR-mediated repression of reporter gene activity (Figure 2B,C). This was consistent with a previous report [8,9], and further questions the notion that AHRR functions by squelching or competing with ARNT thereby reducing the availability of ARNT to interact with AHR at AHREs [4,27]. We previously reported that AHR, but not ARNT overexpression rescued TiPARP-dependent repression of AHR transactivation [15]. Moreover, AHRR [9] and TiPARP [15] directly interact with AHR, suggesting that interactions between AHRR or TiPARP and AHR rather than ARNT are important for repression of AHR. The combination of AHRRΔ8 and TiPARP resulted in significantly more inhibition of CYP1A1 reporter gene levels than either repressor alone. Although these data do not show whether AHRR and TiPARP work together or independently to repress AHR, they suggest that their combined expression results in greater repression of AHR activity (Figure 2D).

### 2.3. TCDD-Induced AHRR Recruitment to AHR-Target Genes

**Figure 3.**
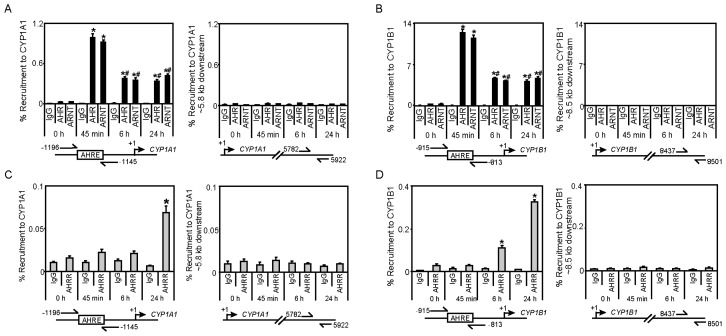
TCDD-induced recruitment of AHR, ARNT and AHRR to *CYP1A1* and *CYP1B1*. Recruitment of (**A**,**B**) AHR, ARNT and (**C**,**D**) AHRR to *CYP1A1* and *CYP1B1* regulatory and distal downstream regions was determined using ChIP assays done on MCF7 cell extracts. Data were presented as means ± SEM from three independent experiments. Recruitment levels significantly different (*p* < 0.05) than 0 h were denoted with an asterisk, whereas recruitment levels significantly different (*p* < 0.05) than 45 min TCDD were denoted with a pound sign.

To determine if endogenous AHRR is recruited to AHR target genes and whether its presence at these genes would affect AHR and ARNT recruitment levels, we examined the TCDD-dependent recruitment of AHR, ARNT and AHRR to *CYP1A1* and *CYP1B1*. Since the recruitment of many transcription factors occurs in a time-dependent manner, chromatin immunoprecipitation (ChIP) assays were done on extracts from MCF7 cells treated with 10 nM TCDD for 45 min, 6 h, and 24 h. TCDD-induced recruitment of AHR and ARNT to *CYP1A1* and *CYP1B1* reached maximum levels after 45 min (Figure 3A,B). Their occupancy levels were reduced by approximately 50% after 6 h and were maintained at those levels at 24 h. No significant differences in AHR or ARNT recruitment to downstream genomic control regions for both genes were observed (Figure 3A,B). TCDD-induced AHRR recruitment to *CYP1A1* was observed only after 24 h TCDD treatment, whereas significant recruitment of AHRR to *CYP1B1* was observed after 6 h and this level was further increased at 24 h (Figure 3C,D). No significant recruitment of AHRR was observed to downstream genomic control regions for both genes (Figure 3C,D). It is worth noting that the antibody used in these experiments does not discriminate between AHRRΔ8 and AHRR_719_, which are both expressed in MCF7 cells, but the AHHRΔ8 is the predominant and active form in this cell line [9]. The time-dependent differences in recruitment of AHRR to *CYP1A1 vs. CYP1B1* could be related to the sensitivity of the ChIP assay, since in our hands the fold increases of AHR or ARNT recruitment to *CYP1B1* in MCF7 cells is always greater than that observed to *CYP1A1* [18,28]. Because we have previously observed TCDD-dependent recruitment of overexpressed TiPARP to the *CYP1A1* [15], these findings together with the data generated here reveal that both AHRR and TiPARP are present at AHR regulatory regions in response to TCDD. Since ChIP assays do not distinguish between direct DNA binding of AHRR or its recruitment to chromatin through tethering to other transcription factors, we cannot conclude that AHRR is recruited to *CYP1A1* or *CYP1B1* through binding to AHREs in their promoter regions. A DNA binding mutant of AHRR represses AHR activity, suggesting that mechanism of AHRR-mediated repression may involve a transrepression mechanism [10]. Future ChIP-Sequencing studies to map the genomic binding patterns of AHRR, and AHRR DNA binding mutant or TiPARP will be important to determine gene selective or DNA sequence preferences between AHRR and TiPARP.

### 2.4. AHRR Knockdown did not Affect TCDD-Mediated AHR Target Transactivation or AHR/ARNT Protein Levels

**Figure 4.**
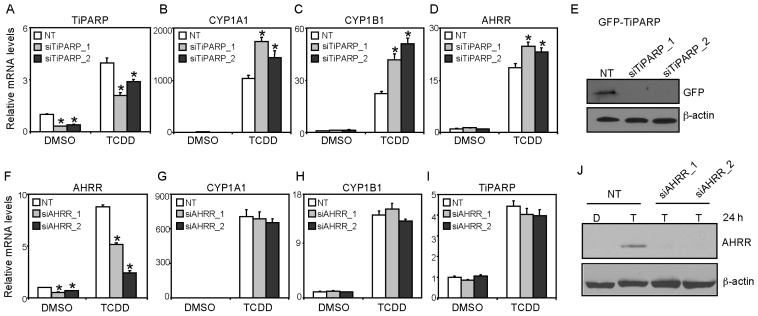
Effect of AHRR or TiPARP knockdown on AHR regulated genes in MCF7 cells. (**A**) TiPARP mRNA expression levels in MCF7 cells following 24 h knockdown with two independent siRNAs targeting TiPARP (siTiPARP_1 and siTiPARP_2) compared with a non-targeting control (NT) siRNA; (**B**–**D**) TiPARP knockdown increased TCDD-induced CYP1A1, CYP1B1 and AHRR mRNA levels after 24 h treatment; (**E**) Immunoblot showing the ability of siRNAs targeting TiPARP (siTiPARP_1 and siTiPARP_2) to reduce GFP-TiPARP protein levels; (**F**) AHRR mRNA expression levels following 24 h knockdown with single siRNAs targeting AHRR (siAHRR_1 and siAHRR_2) compared with NT; (**G**–**I**). AHRR knockdown did not affect TCDD-induced CYP1A1, CYP1B1 and TiPARP mRNA levels after 24 h treatment. Data were presented as means ± SEM of three independent experiments; Expression levels significantly (*p* < 0.05) different than NT were denoted with an asterisk and (**J**) Immunoblot of TCDD-induced AHRR protein expression following RNAi-mediated AHRR knockdown in MCF7 cells. Data shown were from a representative Western blot from three independent experiments.

We next determined the impact of AHRR and TiPARP knockdown on TCDD-induced CYP1A1 and CYP1B1 mRNA levels in MCF7 cells. TiPARP mRNA levels were reduced to approximately 33%–40% compared with non-targeting siRNA (NT) dimethyl sulfoxide (DMSO)-treated cells and resulted in reduced TCDD-dependent increases TiPARP mRNA levels (Figure 4A). Consistent with our previous studies [15], RNAi-mediated knockdown of TiPARP in MCF7 cells significantly increased TCDD-dependent CYP1A1, CYP1B1 and AHRR mRNA levels compared with control cells (Figure 4B–D). So despite increases in AHRR mRNA levels significantly higher CYP1A1 and CYP1B1 expression levels were observed. We confirmed the ability of the siRNAs to decrease TiPARP protein levels by testing the ability of the two different siRNAs to prevent the expression of GFP-TiPARP (Figure 4E). AHRR knockdown reduced AHRR mRNA levels to 40%–45% compared with NT DMSO-treated cells and resulted in reduced TCDD-dependent increases in AHRR mRNA levels (Figure 4F); however, this did not affect constitutive or TCDD-induced CYP1A1, CYP1B1 or TiPARP mRNA levels (Figure 4G–I). Because AHRR protein was not detected in solvent or non-treated MCF7 cells (Figure 1B and data not shown), we tested the ability of the siRNA to target AHRR protein levels after 24 h TCDD treatment. We observed a significant reduction in AHRR protein levels after transfection of both siAHRR sequences compared with TCDD-treated NT cells, suggesting that the failure of AHRR knockdown to affect AHR target gene expression in MCF7 cells was not due to inefficient knockdown at the protein level (Figure 4J).

### 2.5. TiPARP and AHRR Differentially Affect AHR Protein Levels

**Figure 5.**
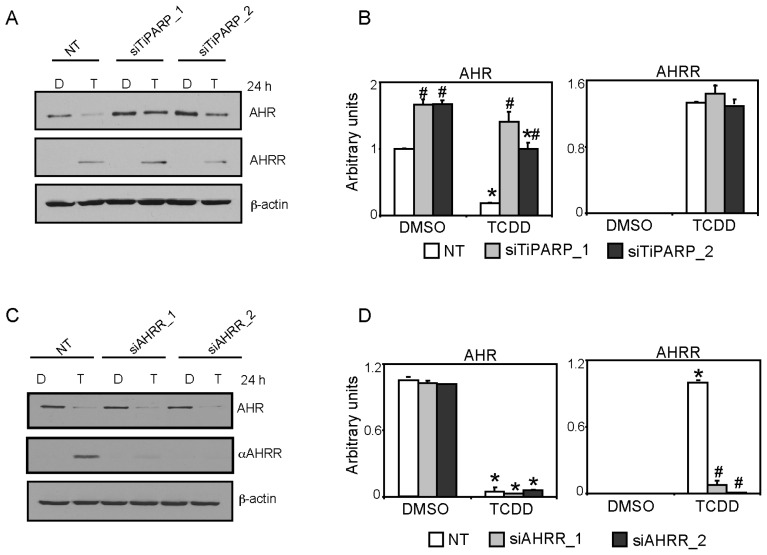
TiPARP, but not AHRR knockdown increases AHR protein levels. (**A**,**B**) TiPARP knockdown increased; while (**C**,**D**) AHRR knockdown did not affect AHR protein levels in MCF7 cells treated for 24 h with dimethyl sulfoxide (DMSO) (**D**) or TCDD (**T**). AHRR protein levels were unaffected by TiPARP knockdown. Data shown were from a representative Western blot from three independent experiments. Quantification of AHR and AHRR protein levels in panels **B** and **D** was done using ImageJ analysis software (NIH). Asterisks denoted protein levels significantly (*p* < 0.05) less than transfection-matched DMSO-treated cells. Pound signs denoted protein levels significantly (*p* < 0.05) different treatment-matched NT cells.

We reported that TiPARP knockdown in T47D cells increased constitutive AHR expression and reduced the TCDD-induced proteasome-mediated degradation of AHR [15]. To determine if this regulation also occurred in MCF7 cells and if AHRR knockdown affected AHR protein levels, we compared AHR and AHRR proteins levels after TiPARP or AHRR knockdown in the presence or absence of TCDD. TiPARP knockdown significantly increased constitutive AHR protein levels and reduced TCDD-dependent AHR degradation compared with NT cells (Figure 5A,B). TiPARP knockdown did not affect the constitutive or TCDD-induced AHRR protein levels (Figure 5A,B). AHRR knockdown did not affect constitutive or TCDD-dependent changes in AHR protein levels (Figure 5C,D). Collectively, these results demonstrate that TiPARP knockdown increased AHR transactivation and AHR protein levels, whereas AHRR knockdown did not alter AHR transactivation nor did it affect AHR protein levels. These findings highlight key differences between the ability of AHRR and TiPARP to repress AHR activity, where TiPARP but not AHRR influences AHR protein levels.

### 2.6. Immortalized AHRR^−/−^ MEFs Exhibit Increased AHR Transactivation but not Increased AHR Protein Levels

**Figure 6.**
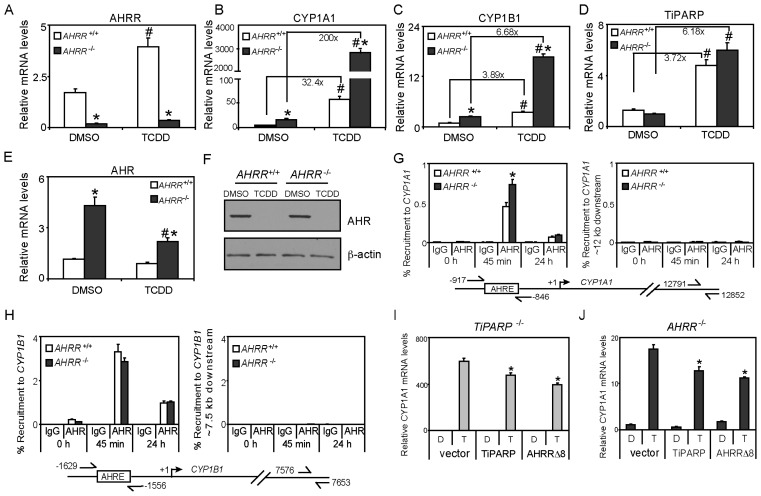
Immortalized *AHRR*^−/−^ mouse embryonic fibroblasts (MEFs) exhibit increased AHR activity. (**A**–**F**) Wildtype (*AHRR*^+/+^) and *AHRR*^−/−^ immortalized MEFs were treated with 10 nM TCDD for 24 h. Gene expression levels were normalized to DMSO treated wildtype cells. Gene expression levels significantly (*p* < 0.05) different than treatment-matched wildtype cells were denoted with an asterisk, and levels significantly (*p <* 0.05) different than genotype-matched cells are denoted with a pound sign; (**H**–**I**) *AHRR*^−/−^ MEFs have similar AHR protein levels as wildtype cells and loss of AHRR did not affect TCDD-induced AHR proteolytic degradation compared with DMSO. Data shown were representative of a Western blot from three independent experiments; (**I**) Quantification of AHR protein levels was done using ImageJ analysis software (NIH). Pound signs denoted AHR protein levels significantly (*p* < 0.05) less than genotype-matched DMSO-treated cells; (**G**) AHR recruitment to *CYP1A1* or to a distal downstream *CYP1A1* region in wildtype or *AHRR*^−/−^ MEFs; (**H**) AHR recruitment to *CYP1B1* or to a distal downstream *CYP1B1* region in wildtype or *AHRR*^−/−^ MEFs. Recruitment levels significantly greater (*p* < 0.05) than treatment-matched wildtype cells were denoted with an asterisk; (**I**,**J**) AHRRΔ8 and TiPARP independently repressed TCDD-induced CYP1A1 mRNA levels. *TiPARP*^−/−^ or *AHRR*^−/−^ MEFs were transfected with pcDNA-AHRRΔ8, pcDNA-mTiPARP (murine TiPARP) or empty vector (pcDNA). Cells were then treated with DMSO or TCDD for 24 h and CYP1A1 mRNA levels were determined. The data were representative of three independent experiments. Gene expression significantly less (*p* < 0.05) than empty vector transfected cells were denoted with an asterisk.

To examine the effect of AHRR loss on AHR transactivation, we generated immortalized MEFs from wildtype (*AHRR*^+/+^) and *AHRR*^−/−^ embryos as described in Materials and Methods. AHRR mRNA levels were significantly lower and were not induced by TCDD in *AHRR*^−/−^ MEFs compared with wildtype cells (Figure 6A). Consistent with its repressor role, constitutive and TCDD-induced CYP1A1 and CYP1B1 mRNA levels were significantly greater in *AHRR*^−/−^ compared with wildtype cells (Figure 6B,C). These findings are in agreement with a previous study reporting that AHRR knockdown in primary fibroblasts restored AHR-dependent regulation of CYP1A1 mRNA levels [20]. However, these data are in contrast to AHRR knockdown studies in MCF7 cells (Figure 4G–I) and may reflect cell-type dependent differences or that the level of AHRR knockdown achieved in MCF7 cells was insufficient to affect AHR target gene expression. We recently generated zinc finger nuclease-mediated AHR or ARNT knockout MCF7 cells [29] and a similar targeted gene knockout strategy could be used for AHRR to further dissect its mechanism of action in human cell lines. Constitutive and TCDD-induced TiPARP mRNA levels were not significantly greater in *AHRR*^−/−^cells (Figure 6D). Increases in TiPARP mRNA levels would be expected since TiPARP, like AHRR, is an AHR target gene and increases in AHR activity due to loss of AHRR would be expected to increase AHR target gene expression. We reported similar increases in AHRR mRNA levels in *TiPARP*^−/−^ MEFs treated with TCDD [15]. However, it is not clear if TiPARP protein levels are also increased in *AHRR*^−/−^ MEFs or if AHRR protein levels are increased in *TiPARP*^−/−^ MEFs. Interestingly, constitutive AHR mRNA levels were significantly higher in *AHRR*^−/−^ MEFs, and these levels were reduced in a TCDD-dependent manner (Figure 6E). This observation is consistent with that observed in primary *AHRR*^−/−^ MEFs [12]. No differences in constitutive AHR protein levels or TCDD-dependent degradation of AHR were observed (Figure 6F). This is in contrast to *TiPARP*^−/−^ MEFs which exhibit higher constitutive AHR proteins levels and reduced TCDD-induced AHR degradation [15]. These data further support that unlike AHRR the mechanism of TiPARP-mediated repression of AHR action involves regulating AHR protein levels. ChIP assays revealed small but significant increases in AHR recruitment to *CYP1A1*, but not *CYP1B1* after 45 min TCDD treatment in *AHRR*^−/−^ MEFs compared with wildtype cells (Figure 6G,H). No differences in AHR occupancy at *CYP1A1* or *CYP1B1* were observed at 24 h (Figure 6G,H). Since we only observed increases in AHR recruitment to *CYP1A1* at 6 h, the increased CYP1A1 and CYP1B1 mRNA levels in TCDD-treated *AHRR*^−/−^ MEFs may be due to increased recruitment of coactivators or reduced recruitment of corepressor proteins to these genes.

We next determined if AHRR repressed AHR transactivation in the absence of TiPARP and *vice versa*. To accomplish this, we overexpressed AHRR in *TiPARP*^−/−^ MEFs and TiPARP in *AHRR*^−/−^ MEFs and determined the effect on TCDD-dependent CYP1A1 mRNA levels. Overexpression of AHRR significantly reduced TCDD-induced CYP1A1 mRNA levels to a similar level, as did overexpression of TiPARP in *TiPARP*^−/−^ MEFs. Similarly, TiPARP or AHRR overexpression significantly reduced CYP1A1 mRNA levels to a similar level in *AHRR*^−/−^ MEFs (Figure 6I,J). These data reveal that TiPARP and AHRR can independently repress AHR transactivation and that the ability of TiPARP to repress AHR regulation of CYP1A1 does not require AHRR and *vice versa*.

### 2.7. AHRR and TiPARP Are Predominantly Nuclear Proteins

**Figure 7.**
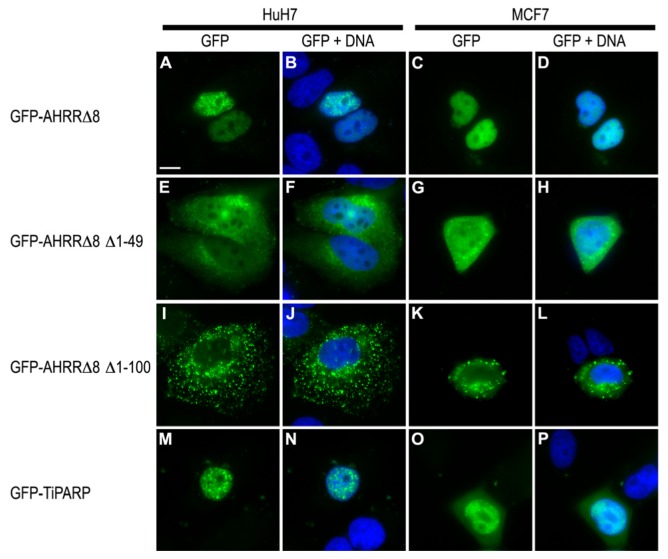
Sub-cellular localization of GFP-AHRRΔ8, GFP-AHRRΔ8 variants and GFP-TiPARP. GFP-AHRRΔ8 (green) was localized in the nucleus (blue) of HuH7 and MCF7 cells (**A**–**D**); GFP-AHRRΔ8_Δ1-49 was present in both the nucleus and the cytoplasm (**E**–**H**); while GFP-AHRRD8_Δ1-100 was localized predominantly in large foci in the cytoplasm (**I**–**L**); Wild-type GFP-TiPARP was localized in the nucleus in a pattern similar to that of GFP-AHRRΔ8, although faint cytoplasmic expression was observed in MCF7 cells (**M**–**P**). Scale bar represents 10 mm (**A**); all panels were to same scale. Data shown were representative images from three independent experiments.

AHRR has been previously described to undergo nucleocytoplasmic shuttling, with an equilibrium favoring nuclear localization [30]. Other labs have reported the nuclear localization of AHRR, but there are no reports of the nuclear localization of AHRRΔ8. TiPARP is also primarily localized to the nucleus, although it has also been reported to exhibit cytosolic staining in Hela cells [31]. To gain insight into the mechanism of action of AHRR and TiPARP, we determined the expression patterns of AHRRΔ8 and TiPARP in MCF7 and HuH7 cells. GFP-AHRRΔ8 localized in the nucleus in both HuH7 and MCF7 cells as diffuse nuclear foci (Figure 7A–D). Similar findings were observed for GFP-AHRR_719_ (data not shown). The nuclear localization sequence (NLS) of AHRR was reported to map to amino acids 16-RKRRR-20, and unlike the NLS of AHR it consisted of a monopartite rather than a bipartite sequence [30]. To test whether AHRRΔ8 lacking its putative NLS sequence would exhibit cytosolic staining in both cell lines, we constructed a GFP-AHRRΔ8_Δ49 and GFP-AHRRΔ8_Δ100 in which the first 49 containing the putative NLS or first 100 amino acids were removed, respectively. GFP-AHRRΔ8_Δ1-49 was present in both the nucleus and the cytoplasm in HuH7 and MCF7 cells (Figure 7E–H), whereas GFP-AHRRΔ8_Δ1-100 localized predominantly as large foci in the cytoplasm (Figure 7I–L). This indicates that there may be additional sequences between amino acids 50–99 that influence the nuclear localization of AHRR or that AHRR contains a bipartite rather than monopartite sequence. GFP-TiPARP exhibited a similar nuclear expression patterns to that of GFP-AHRRΔ8 in HuH7 and MCF7 cells, although faint and diffuse cytoplasmic staining was observed in MCF7 cells (Figure 7M–P).

**Figure 8.**
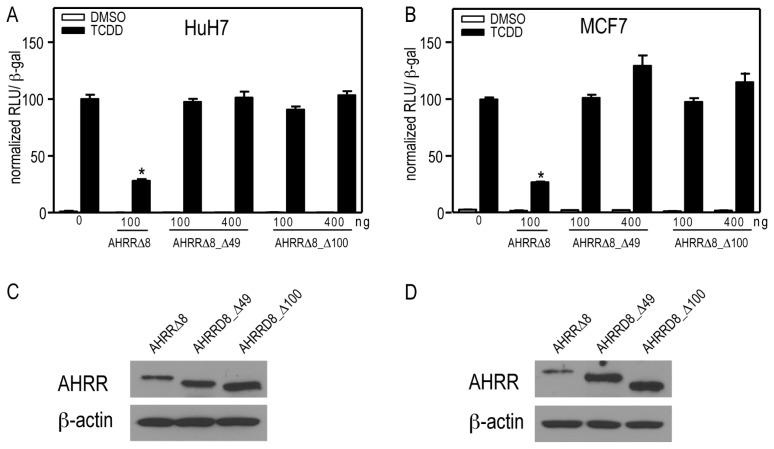
AHRRΔ8_Δ1-49 and AHRRΔ8_Δ1-100 do not repress CYP1A1-regulated reporter gene activity in HuH7 or MCF7 cells. (**A**) HuH7 or (**B**) MCF7 cells were transfected with pCYP1A1-luc and pcDNA-AHRRΔ8 or increasing concentrations of pcDNA-AHRRΔ8_Δ1-49 and pcDNA-AHRRΔ8_Δ1-100. Cells were then treated with TCDD for 24 h and reporter gene activity determined. The data are presented as means ± SEM of three independent replicates. Reporter gene activity significantly less (*p* < 0.05) than cells transfected AHRRΔ8 is denoted by an asterisk. Protein expression levels of AHRRΔ8 and AHRRΔ8 variants in transfected (**C**) HuH7 or (**D**) MCF7 cells was determined by Western blotting. Data shown were representative of three independent experiments.

We next determined whether GFP-AHRRΔ8_Δ49 and GFP-AHRRΔ8_Δ100 were able to repress AHR-dependent CYP1A1 reporter gene activity in transfected HuH7 cells. Both GFP-AHRRΔ8_Δ49 and GFP-AHRRΔ8_Δ100 failed to repress CYP1A1 reporter gene activity (Figure 8A,B). Western blots showed that the lack of repression was not due to a lack of expression of either truncated AHRRΔ8 mutant (Figure 8C,D). These data reveal that amino acid residues 1–49 are required for AHRR-dependent repression and that the nuclear form of GFP-AHRRΔ8_Δ49 is unable to inhibit AHR under our assay conditions. This is most likely not due to the lack of DNA binding because a DNA binding mutant of AHRR still represses AHR [10], but may be due to reduced interaction with AHR or other accessory proteins that are required for this inhibition.

## 3. Experimental Section

### 3.1. Chemicals and Biological Reagents

2,3,7,8-tetrachlorodibenzo-*p*-dioxin (TCDD) was purchased from Wellington Laboratories (Guelph, ON, Canada). Cell culture media, fetal bovine serum (FBS), trypsin-EDTA and dimethyl sulfoxide (DMSO) were purchased from Sigma-Aldrich (St. Louis, MO, USA). All other chemicals and biological reagents were purchase from other vendors and were of the highest quality.

### 3.2. Plasmids

The pcDNA-AHRRΔ8 (delta exon 8) and pcDNA-AHRR were generous gifts from Dr. Mark Hahn (Woods Hole Oceanographic Institution, Wood’s Hole, MA, USA). Both of these plasmids have been described elsewhere [9]. To create pEGFP-AHRRΔ8 we first PCR amplified AHRRΔ8 using primers containing EcoRI and XhoI. The plasmid EGFP-AHRRΔΔ49 by PCR amplifying amino acids 50–701 of AHRRΔ using primers 5′-CAAAGAATTCGCCGAGTTGGACCACCTG-3′ and 5′-CAAACTCGAGCTATGGCAGGAATGTGCACCCC-3′ and then digested with EcoRI and XhoI. The plasmid EGFP-AHRRΔ100 by PCR amplifying amino acids 101–701 of AHRR 00 by PCR amplif′-CAAAGAATTCTCGCCCGGAGACAGCTGT-3′ and the same reverse primer as above and then digested with EcoRI and XhoI. The digested PCR products were then ligated into EcoRI and SalI sites of pEGFP.

### 3.3. Cell Culture

MCF7 human breast carcinoma cells were purchased from ATCC (Manassas, VA, USA). HuH7 human hepatoma cells were a kind gift from Dr. Jan-Ake Gustafsson (University of Houston, Houston, TX, USA). The derivation and characterization of the *TiPARP*^−/−^ mouse embryonic fibroblasts (MEFs) have been described elsewhere [15]. Primary *AHRR*^−/−^ and wildtype MEFs were immortalized after transfection with Simian virus large T antigen and a puromycin resistance plasmid, and selected in puromycin-containing medium for 1 week [12]. MCF7 cells were cultured in low-glucose (1 g/L) DMEM (Dulbecco’s modified Eagle’s media) supplemented with 10% (*v*/*v*) FBS and 1% (*v*/*v*) penicillin/streptomycin (PEST). HuH7 and all MEF cells were cultured in high glucose (4.5 g/L) DMEM supplemented with 10% FBS and 1% PEST. All cell lines were maintained at 37 °C and 5% CO_2_ and subcultured every 2–3 days or when cells reached 80% confluency.

### 3.4. Transient Transfection, Reporter Gene and RNAi Studies

For the reporter gene assays, HuH7 cells were transfected with or 500–1000 ng pcDNA-AHRR or 5–100 ng pcDNA-AHRRΔ8 or 100 ng pcDNA-TiPARP [15] or co-transfected with 5 or 100 ng pcDNA-AHRRΔ8 and 100–500 ng pCMV-AHR or 100–500 ng pcDNA4-ARNT [32] using Lipofectamine LTX (Invitrogen Canada, Burlington, ON, Canada). All reactions included 50 ng of pCH110-β-Gal to normalize for transfection efficiency. After 24 h cells were treated with TCDD or DMSO for 24 h before luciferase and β-galactosidase assays were c. For the RNAi studies, AHRR siRNAs (siAHRR_1; SASI_Hs01_00213384, siAHRR_2; SASI_Hs02_00353811) were purchased from Sigma-Aldrich. TiPARP (siTiPARP_1; J-013948-12-0005, siTiPARP_2; J-013948-13-0005) ON-TARGETplus siRNAs and DharmaFECT1 transfection reagent were purchased from Dharmacon (Lafayette, CO, USA). MCF7 cells were transfected with 2 μL DharmaFECT1 and 50 nM of each siRNA or non-targeting siRNA #2 (NT; D-001810-02-20) (Dharmacon). Twenty-four hours after transfection the cells were treated with 10 nM TCDD or DMSO (0.1%) for 24 h.

Immortalized *AHRR*^−/−^ and *TIPARP*^−/−^ MEFs were transiently transfected by nucleofection according to manufacturer’s instructions (Amaxa Inc., Gaithersburg, MD, USA). Briefly 1 × 10^6^ *AHRR*^−/−^ MEFs or 2 × 10^6^ *TiPARP*^−/−^ MEFs were electroporated with 10 μg pcDNA-mTIPARP, 5 μg pcDNA-AHRRΔ8 in 100 μL nucleofector solution using A023 program on nucleofector device. The following day, the transfected cells were treated with DMSO or TCDD for 24 h. RNA was isolated and reverse transcribed as described below.

### 3.5. RNA Isolation and qPCR

MCF7 cells were seeded in 6-well plates 24 h prior to treatment with 10 nM TCDD for up to 24 h. MEFs were seeded in 6-well plates at a density of 100,000 cells/well. The following day cells were treated with 10 nM TCDD or DMSO for 24 h. RNA was isolated with Aurum Total RNA Mini Kit (Bio-Rad, Mississauga, ON, Canada) and reverse transcribed using SuperScriptIII (Invitrogen Canana, Burlington, ON, Canada). QPCR was done with 1 μL of the cDNA synthesis reaction using SsoFast EvaGreen SYBR Supermix (Bio-Rad). Primers used to amplify human AHRR mRNA were 5′-CCCCGCCCTTGGAGACAGGA-3′ and 5′-AGTACTCGGTGGGCGTGCCT-3′. Primers for human CYP1A1, CYP1B1 and TiPARP mRNAs have been previously described [28,33]. Primers for mouse AHRR mRNA were 5′-CTGCCCAGGTACTCTGAACC-3′ and 5′-ACTGTCCACAAAGCCTGACC-3′; for CYP1A1 mRNA were 5′-CGTTATGACCATGATGACCAAGA-3′ and 5′-TCCCCAAACTCATTGCTCAGAT-3′; for CYP1B1 mRNA were 5′-CCAGATCCCGCTGCTCTACA-3′ and 5′-TGGACTGTCTGCACTAAGGCTG-3′; for TIPARP mRNA were 5′-CCTTCAACCAAGAACAAAGCATCT-3′; and for AHR mRNA were 5′-AGCATCATGAGGAACCTTGG-3′ and 5′-GGATTTCGTCCGTTATGTCG-3′. All target transcripts were normalized to β-actin and analyzed using the comparative *C*_t_ (ΔΔ *C*_t_) method. RNA data were presented as mean and standard error of the mean of three independent experiments.

### 3.6. Western Blot

Whole cell extracts were prepared from transfected cells treated with 10 nM TCDD or DMSO and proteins were resolved by SDS-PAGE and transferred to nitrocellulose membrane. The membrane was blocked in 2% (*w*/*v*) fat-free milk PBS/T for 1 h at room temperature and then incubated with anti-AHR (H-211, Santa Cruz, Dallas, TX, USA), anti-ARNT antibody (C-19, Santa Cruz), anti-AHRR antibody (HPA019614, Sigma-Aldrich) or anti-β-actin antibody (Sigma) overnight at 4 °C. After washing, the bands were visualized using ECL Prime chemiluminescent substrate (GE Healthcare, Baie d’Urfe, QC, Canada) or SuperSignal West Dura substrate (Pierce, Rockford, IL, USA) according to the manufacturer’s instructions. For the immortalized MEFs, each cell line was plated at a density of 2 million cells in 10 cm dishes. The following day, cells were treated with 10 nM TCDD or DMSO for 24 h, whole cell extracts were prepared and proteins resolved as described above. Membranes were incubated with anti-AHR (SA-210, Enzo Life Sciences Inc., Farmingdale, NY, USA) overnight and incubated 1 h with anti-rabbit HRP-conjugated secondary antibody. Quantification of protein bands was done with ImageJ software (NIH) and they were normalized to β-actin levels.

### 3.7. Chromatin Immunoprecipitation

MCF7 cells were plated in 10 cm dishes in culture media at a density of 3.5 × 10^6^ cells/dish and treated with 10 nM TCDD for 45 min, 6 h and 24 h and ChIP assays were done as previously described [34] using 1 μg of anti-AHR (H-211) or anti-ARNT (H-172). Isolated DNA was quantified by qPCR using SsoFast EvaGreen SYBR Supermix. Primers used to amplify *CYP1A1* and *CYP1B1* have been previously described [33]. For immortalized wildtype and *AHRR*^−/−^ MEFs, each cell line was plated at a density of 5 × 10^5^ cells per 10 cm dish. The following day cells were treated with 10 nM TCDD or DMSO at the indicated times and ChIP assays were done using 1 μg anti-AHR (SA-210) antibody. Primers used to amplify mouse *CYP1A1* and *CYP1B1* regulatory regions have been previously described [35]. Data were reported as percentages relative to 100% total input chromatin.

### 3.8. Indirect Immunofluorescence

MCF7 or HuH7 cells were seeded onto glass cover slips in a 6-well plate (3.5 × 10^5^ cells/well). HuH7 cells were transfected with 100 ng of GFP-AHRR_Δ8, GFP-AHRRΔ8_Δ49, GFP-AHRRΔ8_Δ101, or 500 ng of GFP-TiPARP using lipofectamine 2000. After 24 h, cells were fixed with 4% paraformaldehyde, permeabilized with 0.4% Triton X-100, and mounted onto slides with Vectashield mounting medium containing DAPI to stain DNA (Vector Laboratories, Burlingame, CA, USA). GFP-expressing cells were visualized using a Zeiss AxioImager M2 epifluorescence microscope and images were captured with a Zeiss AxioCam MRm camera and Zeiss AxioVision software.

### 3.9. Statistical Analysis

All data were presented as means and standard error of the mean. A one-way Analysis of Variance (ANOVA) followed by Tukey’s multiple comparison tests was used to assess statistical significance (*p* < 0.05).

## 4. Conclusions

In this study, we compared the ability of AHRR and TiPARP to repress AHR activity and regulate AHR protein levels. AHRR and TiPARP exhibit a number of similarities including that their expression is increased by ligand activated AHR, that they directly interact with AHR, and that increased ARNT expression does not affect their ability to repress AHR [15]. There are, however, also some notable differences. For example, TiPARP overexpression increases the proteolytic degradation of AHR, suggesting that TiPARP may act as a more general regulator of AHR activity [15]. Moreover, knockdown of TiPARP in a number of cell lines results in increased AHR target gene expression [15]. However, in a chicken embryo model, TiPARP was reported to exacerbate TCDD toxicity by reducing phosphoenolpyruvate carboxykinase expression levels and consequently reducing gluconeogenesis [36,37]. However, unlike human and mouse TiPARP, overexpression of chicken (*Gallus gallus*) TiPARP in HuH7 cells does not repress AHR regulated reporter gene activity [15]. These data suggest that TiPARP’s ability to repress AHR exhibits species or context specificity. Careful analysis of *TiPARP*^−/−^ mice will be important to determine the impact of TiPARP loss on AHR signaling pathways and TCDD sensitivity. Studies of *TiPARP*^−/−^ mice treated with TCDD or other AHR ligand will also help dissect the overlapping and independent mechanisms through which AHRR and TiPARP repress AHR, solidifying TiPARP and AHRR as key negative regulators and important components of the AHR signaling pathway in human and mouse cell lines.
